# Supplementary material for: The Control of Metabolic CO2 in Public Transport as a Strategy to Reduce the Transmission of Respiratory Infectious Diseases
Source: Int J Environ Res Public Health. 2022 May 28;19(11):6605. doi: 10.3390/ijerph19116605 (PMC9180361; doi:10.3390/ijerph19116605)
Supplement: Supplementary file 1 [file ijerph-19-06605-s001.zip › ijerph-1722322-supplementary.pdf]

Measurement of metabolic CO_2 in collective transport: a method to reduce the spread of respiratory infectious diseases

Marta Baselga-Lahoz[1], Juan J. Alba[1,2], Alberto J. Schuhmacher[1,3]

[1] Institute for Health Research Aragon (IIS Aragón), 50009 Zaragoza, Spain

[2] Department of Mechanical Engineering, University of Zaragoza, Campus Río Ebro – I+D Building, 50018 Zaragoza, Spain

[3] Fundación Agencia Aragonesa para la Investigación y el Desarrollo (ARAID), 500018 Zaragoza, Spain

Supplementary Material Section

Table S1. Weekend journeys data analysis (from Friday evening to Sunday).

	**Morning journeys (8:50h – 11:30 h)**										
	#1	#2	#3	#4	#5						
**Average (ppm)**	695	771	572	642	708						
**Standard Deviation (ppm)**	140	196	75	158	191						
**Maximum (ppm)**	929	1112	701	1017	1079						
**Minimum (ppm)**	464	486	464	460	445						
	**Afternoon journeys (11:31 h – 16:30 h)**										
	#5	#6	#7	#8	#9	#10	#11	#12	#13	#14	#15
**Average (ppm)**	715	753	641	720	835	790	644	728	634	541	595
**Standard Deviation (ppm)**	162	190	153	169	232	235	137	181	152	82	116
**Maximum (ppm)**	1011	1133	1012	1011	1229	1249	884	1032	913	704	800
**Minimum (ppm)**	476	485	465	483	486	475	471	469	444	436	445
	**Evening journeys (16:31 h – 20:30 h)**										
	#16	#17	#18	#19	#20	#21	#22	#23	#24		
**Average (ppm)**	701	685	788	709	680	696	675	674	614		
**Standard Deviation (ppm)**	186	177	237	198	187	250	161	180	142		
**Maximum (ppm)**	1071	999	1198	1097	1059	1215	955	1094	880		
**Minimum (ppm)**	469	465	469	470	471	454	457	448	451		

Table S2. Weekday journeys data analysis (from Monday to Friday evening).

	**Morning journeys (8:50h – 11:30 h)**							
	#25	#26	#27	#28	#29	#30	#31	
**Average (ppm)**	787	675	647	720	648	662	736	
**Standard Deviation (ppm)**	227	162	129	175	159	139	188	
**Maximum (ppm)**	1159	993	956	1030	996	944	1058	
**Minimum (ppm)**	466	472	501	499	481	491	479	
	**Afternoon journeys (11:31 h – 16:30 h)**							
	#32	#33	#34	#35	#36	#37	#38	#39
**Average (ppm)**	715	753	720	685	671	629	748	709
**Standard Deviation (ppm)**	162	190	175	160	142	147	187	183
**Maximum (ppm)**	1011	1133	1030	954	996	919	1121	1091
**Minimum (ppm)**	476	485	499	470	471	451	472	486
	**Evening journeys (16:31 h – 20:30 h)**							
	#40	#41	#42	#43	#44			
**Average (ppm)**	708	705	604	600	648			
**Standard Deviation (ppm)**	188	170	97	102	125			
**Maximum (ppm)**	1090	964	794	805	843			
**Minimum (ppm)**	468	480	475	476	478			
